# Enhancement of cell proliferation in various mammalian cell lines by gene insertion of a cyclin-dependent kinase homolog

**DOI:** 10.1186/1472-6750-7-71

**Published:** 2007-10-18

**Authors:** Pratik Jaluria, Michael Betenbaugh, Konstantinos Konstantopoulos, Joseph Shiloach

**Affiliations:** 1 Department of Chemical and Biomolecular Engineering, Johns Hopkins University. 221 Maryland Hall, 3400 North Charles Street, Baltimore, MD 21218 USA; 2 National Institute of Diabetes & Digestive & Kidney Diseases, National Institutes of Health, Biotechnology Unit, Building 14A, Room 170, Bethesda, MD 20892, USA

## Abstract

**Background:**

Genomics tools, particularly DNA microarrays, have found application in a number of areas including gene discovery and disease characterization. Despite the vast utility of these tools, little work has been done to explore the basis of distinct cellular properties, especially those important to biotechnology such as growth. And so, with the intent of engineering cell lines by manipulating the expression of these genes, anchorage-independent and anchorage-dependent HeLa cells, displaying markedly different growth characteristics, were analyzed using DNA microarrays.

**Results:**

Two genes, cyclin-dependent kinase like 3 (*cdkl3*) and cytochrome c oxidase subunit (*cox15*), were up-regulated in the faster growing, anchorage-independent (suspension) HeLa cells relative to the slower growing, anchorage-dependent (attached) HeLa cells. Enhanced expression of either gene in the attached HeLa cells resulted in elevated cell proliferation, though insertion of *cdkl3 *had a greater impact than that of *cox15*. Moreover, flow cytometric analysis indicated that cells with an insert of *cdkl3 *were able to transition from the G0/G1 phases to the S phase faster than control cells. In turn, expression of *cox15 *was seen to increase the maximum viable cell numbers achieved relative to the control, and to a greater extent than *cdkl3*. Quantitatively similar results were obtained with two Human Embryonic Kidney-293 (HEK-293) cell lines and a Chinese Hamster Ovary (CHO) cell line. Additionally, HEK-293 cells secreting adipocyte complement-related protein of 30 kDa (acrp30) exhibited a slight increase in specific protein production and higher total protein production in response to the insertion of either *cdkl3 *or *cox15*.

**Conclusion:**

These results are consistent with previous studies on the functionalities of *cdkl3 *and *cox15*. For instance, the effect of *cdkl3 *on cell growth is consistent with its homology to the *cdk3 *gene which is involved in G1 to S phase transition. Likewise, the increase in cell viability due to *cox15 *expression is consistent with its role in oxidative phosphorylation as an assembly factor for cytochrome c oxidase and its involvement removing apoptosis-inducing oxygen radicals. Collectively, the present study illustrates the potential of using microarray technology to identify genes influential to specific cellular processes with the possibility of engineering cell lines as desired to meet production needs.

## Background

The properties of a cell line being used to produce therapeutic or diagnostic compounds are vital elements in designing a production process [1-3]. Such properties include: nutritional requirements, adhesion capabilities, glycosylation capabilities, and cellular response to stimuli [1,2]. Perhaps one of the most important cellular properties is proliferation, referring to the rate at which cells grow and divide by passing through the different cell cycle phases [3,4].

The ability to alter specific properties of a cell line would undoubtedly find application in the field of biotechnology. Currently, protocols and technical notes are readily available to adapt specific cell lines to make them more suitable for a particular process. These procedures have focused primarily on screening cell populations to identify and subsequently select cells with the desired feature [3-5]. Less emphasis has been placed on either identifying how a particular adaptation occurs or the ability to engineer the desired modification directly into the cell lines.

Previous studies exploring proliferation have examined the roles of various genes and/or chemicals regulating the cell cycle in hopes of indirectly stimulating cellular growth [6,7]. For example, it was shown that transfection of the gene *c-myc*, a proto-oncogene that encodes a transcription factor, can enhance the growth rate of Chinese Hamster Ovary (CHO) cells [8]. Other studies have focused on deciphering the sequence of events necessary for cells to complete the cell cycle by targeting specific genes [1,9]. It has been found that certain proteins are necessary for cells to maintain viability and/or function such as growth factors and stress sensors [10]. Some studies have identified groups or families of proteins that play important roles maintaining cellular function such as members of the serine/threonine protein kinase family; essential for phase transitions of the cell cycle [10]. Based on this, a number of studies have explored what other proteins associate with members of this kinase family such as cyclins, inhibitors, and ubiquitin [4,10]. However, the complexity of the cell cycle has limited the way in which mammalian cells can be manipulated to increase their growth.

In the present study, complementary DNA (cDNA) microarrays were used to identify genes potentially influencing cellular growth by comparing a slow growing, anchorage-dependent (attached) cell line to a fast growing, anchorage-independent (suspension) cell line. Both cell lines were HeLa cell lines [11]. Microarray data were probed for differentially expressed genes using clustering algorithms and proposed functionalities. Two genes, *cdkl3 *[GenBank: NM016508] and *cox15 *[GenBank: NM078470], had higher expression in the fast growing, suspension HeLa cells than in the slow growing, attached HeLa cells. The cDNA for both genes were transfected into a number of cell lines including Human Embryonic Kidney-293 (HEK-293), CHO, and Madin-Darby Canine Kidney (MDCK). The growth characteristics of these cell lines were monitored along with recombinant protein production levels in the HEK-293 ACRP30 (adipocyte complement-related protein of 30 kDa) cell line using ELISA. Further characterization of the transfected cell physiology was performed using flow cytometry analysis.

## Results

### 1. Growth of HeLa cells in bioreactors

**Figure 1:**
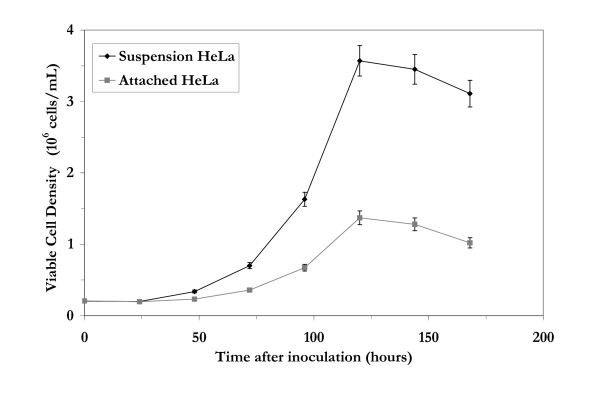
Growth of suspension and attached HeLa cells in bioreactors under batch conditions. Attached HeLa cells were grown on Cytodex 3 microcarriers.

The attached and suspension HeLa cell lines were grown in bioreactors in three independent experiments using the same media and culture environment. The attached HeLa cells had to be grown using microarriers [12]. The measured viable cell densities for both cells lines are shown in Figure 1. The suspension HeLa cells grew at a maximum specific growth rate of 0.038 ± 0.002 h^-1^, 40% higher than the attached HeLa cells which grew at a maximum specific growth rate of 0.027 ± 0.001 h^-1^. The suspension HeLa cells achieved a maximum viable cell density of about 3.5 × 10^6 ^cells/mL, twice the maximum viable cell density obtained for the attached HeLa cells. The overall viability for each cell line was above 90% until the stationary phase of growth was reached.

### 2. Comparison of gene transcription levels between suspension and attached HeLa cells

Samples for microarray analysis were taken at the same growth phases from each bioreactor, at regular intervals. Imaging software was used to determine which comparisons exhibited the most significant differences between the two cell lines and had the highest levels of overall transcriptional activity. Samples from the middle exponential growth phase, corresponding to the maximum specific growth rates, were found to meet these criteria. This information was deciphered by examining scanned images of hybridized microarrays using GenePix, a visualization software program.

Analysis of the hybridized cDNA microarrays began with quality control (i.e. removing spots of poor signal) and continued with implementation of total intensity normalization [13,14]. The data were filtered by removing genes lacking consistency between slides in terms of how close the expression ratio from any one slide was to the median expression ratio calculated from all of the slides. The data were also screened for genes with expression ratios from the slides in the same direction (i.e. either upregulated or downregulated across all of the slides).

**Figure 2:**
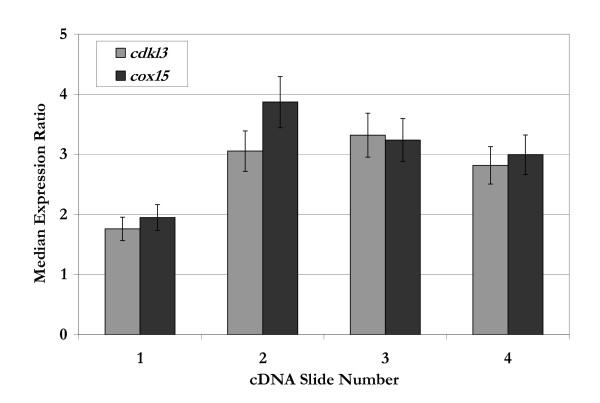
Median expression ratios for *cdkl3 *and *cox15 *from four different spotted cDNA microarray slides. The median expression ratio is calculated from 3 or more gene-specific spots on a single slide. The error bars indicate the range in expression ratios observed for a given slide.

Using clustering algorithms, the genes were organized into groups [14,15]. Principle component analysis (PCA) and gap statistic were used to estimate the number and size of groups inherently present in the data. Results of these algorithms indicated groups of 8, 9, 13, or 14 were likely to form. With this information, self-organizing maps (SOMs) and hierarchical clustering were then applied to the data to segregate the genes into distinct groups. These clusters were probed to identify subsets of genes with relevance to cellular growth based on known or proposed functionalities related to cell cycle regulation, apoptosis, and/or signal transduction. Genes that could be categorized in this manner were then interrogated further based on the level of differential expression with a sampling of the results shown in Table 1. The ratios listed in Table 1 were determined from a sample size of at least 12; based on the number of spots per slide and the number of slides analyzed. Of the genes listed, *cdkl3 *[GenBank: NM016508] and *cox15 *[GenBank: NM078470] were selected for further analysis. As shown in Figure 2, both *cdkl3 *and *cox15 *had expression levels greater than 1, indicating higher expression in the suspension HeLa cell line than in the attached HeLa cell line.

**Table 1:** Gene names and maximum/minimum expression ratios for differentially expressed genes identified following normalization and application of clustering algorithms. Expression ratios are of the form: suspension HeLa/attached HeLa

Gene Symbol	Gene Name	Maximum Expression Ratio	Minimum Expression Ratio
***cdkl3***	cyclin-dependent kinase-like 3	3.32	1.76
***cox15***	cytochrome c oxidase assembly protein	3.87	1.95
***agpat2***	1-acylglycerol-3-phosphate O-acyltransferase 2	3.13	1.64
***fgf7***	Fibroblast growth factor 7	3.50	1.75
***dtymk***	deoxythymidylate kinase (thymidylate kinase)	4.05	1.92
***arhf***	ras homolog gene family f	2.95	1.70
***plagl1***	pleiomorphic adenoma gene-like 1	0.04	0.02
***tial1***	cytotoxic granule-associated RNA binding protein-like 1	0.68	0.14

Note: Each cDNA slide analyzed had at least 3 spots for each of the genes listed above. Analysis of 4 slides translated to a total of at least 12 ratios for each gene. Both the maximum and the minimum expression ratios per gene were determined from these 12 ratios (i.e. sample size).

### 3. Verification of microarray results

As can be seen in Table 1 and Figure 2, the expression ratios of both *cdkl3 *and *cox15 *varied over the different samples, establishing the need to verify these results. For this purpose, a series of RT-PCR experiments were conducted, the results of which are shown in Table 2 along with two controls,*gapd *and *pgk1*. The findings in the Table demonstrate the expression levels are consistent between the microarray data and the RT-PCR experiments.

**Table 2:** Median expression levels for specific genes as determined by cDNA microarrays and RT-PCR. Expression ratios are of the form: suspension HeLa/attached HeLa

Gene Symbol	Gene Name	cDNA microarrays	RT-PCR
***cdkl3***	cyclin-dependent kinase-like 3	2.74	2.6 ± 0.2
***cox15***	cytochrome c oxidase assembly protein	3.01	3.4 ± 0.3
***gapd***	Glyceraldehyde-3-phosphate dehydrogenase	1.03	1.2 ± 0.1
***pgk1***	Phosphoglycerate kinase 1	0.98	1.0 ± 0.1

### 4. Enhanced expression of cdkl3 and cox15 in attached HeLa cells

**Figure 3:**
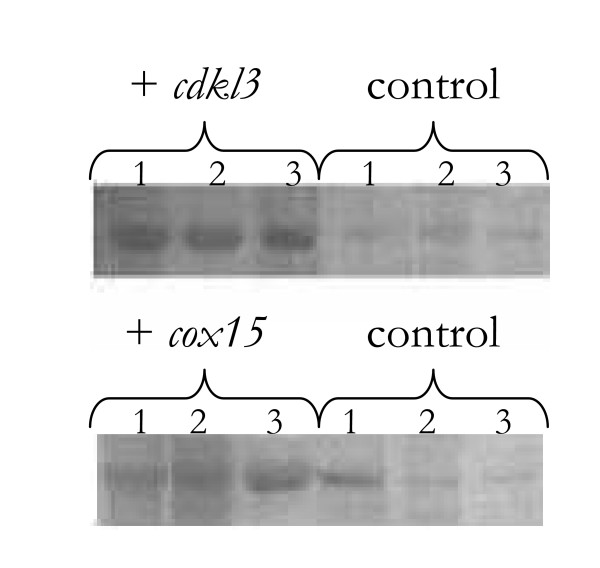
Western blot analysis for several different cell lines indicating relative expression levels. These cells were derived from 3 different colonies that had already been screened and selected for expression of the transfected plasmid. At the time of analysis, these cells had been grown for over 6 days, post-transfection. The control cells were transfected with blank plasmids. 1 – HeLa 2 – HEK-293 3 – CHO

To evaluate the impact of these two genes in cell culture, DNA plasmids containing either *cdkl3 *or *cox15 *were transfected into the attached HeLa cells. Cells transfected with only blank plasmids (i.e. containing neither *cdkl3 *nor *cox15*) served as the control. Each plasmid also contained the neomycin gene allowing cells to be selected based on resistance to geneticin. A number of clones were selected in this manner over the course of 1–2 weeks (post-transfection) and then assayed for expression of the translated protein using western blots. Figure 3 illustrates the protein expression of cells that have gone through this selection process. These blots indicate cells transfected with either *cdkl3 *or *cox15 *had significantly higher protein levels than the control cells. It should be noted that when the selection marker is not present in the culture media, these cell lines return to normal protein production levels within 5–6 passages.

The growth profiles/characteristics of the attached HeLa cells transfected with *cdkl3*, *cox15*, or a blank plasmid are summarized in Table 3. All three cell lines were grown on microcarriers in 250 mL spinner flasks. The maximum specific growth rates, determined in three independent experiments, revealed that the attached HeLa cells transfected with a plasmid containing either *cdkl3 *or *cox15 *grew faster than the control cells. Cells expressing *cdkl3 *had a maximum specific growth rate 20% greater than the control cells while the cells expressing *cox15 *had a maximum specific growth rate 15% greater than the control cells. Using a t-test, it was determined that the differences in maximum specific growth rates between treated and control cells were statistically significant for α-values, also referred to as risk level (i.e. the chance associated with finding a statistically significant difference between means when there is none) as low as 0.005. It was also observed that the duration of the lag phase in cells expressing either *cdkl3 *or *cox15 *was 5 – 8% shorter than that of the control cells.

**Table 3:** Growth-related data for varying cell lines grown in spinner flasks

Cell Type	Cell line	Maximum growth rate during the exponential growth phase (hours^-1^) from 3 different runs				Maximum viable cell density (10^6 ^cells/mL)
		Run 1	Run 2	Run 3	Average Change vs. Control (%)	

HeLa	control	0.027	0.026	0.026		1.49 ± 0.03
	+ *cdkl3*	0.031	0.032	0.032	20	1.53 ± 0.03
	+ *cox15*	0.030	0.030	0.031	15	1.66 ± 0.02
HEK-293	control	0.030	0.030	0.032		1.98 ± 0.02
	+ *cdkl3*	0.035	0.037	0.037	19	2.04 ± 0.04
	+ *cox15*	0.034	0.036	0.034	13	2.15 ± 0.05
HEK-293 ACRP30	control	0.031	0.031	0.032		1.94 ± 0.04
	+ *cdkl3*	0.037	0.037	0.035	16	2.01 ± 0.02
	+ *cox15*	0.036	0.035	0.036	14	2.10 ± 0.06
CHO	control	0.033	0.034	0.034		1.95 ± 0.01
	+ *cdkl3*	0.036	0.037	0.037	9	2.04 ± 0.02
	+ *cox15*	0.036	0.035	0.035	5	2.09 ± 0.03
MDCK	control	0.029	0.030	0.030		1.12 ± 0.04
	+ *cdkl3*	0.030	0.029	0.030	0	1.10 ± 0.02
	+ *cox15*	0.028	0.029	0.028	-5	1.08 ± 0.07

Cells expressing *cox15 *were also able to achieve a maximum viable cell density 11% higher than the control cells whereas cells expressing *cdkl3 *achieved a maximum viable cell density equivalent to the control cells (Table 3). The difference between cells expressing *cox15 *and control cells were statistically significant for α-values as low as 0.005. Similar results, in terms of growth rates and cell densities, were obtained when these cell lines were grown in T-flasks. No additive effects were observed when both genes were simultaneously expressed (data not shown).

### 5. Gene sequence homology and enhanced expression of cdkl3 and cox15 in other cell lines

Using two online databases, Harvester and GenBank, the nucleotide sequences for both *cdkl3 *and *cox15 *were determined [16,17]. Sequence analysis was performed to quantify homology between varying species. For the *cdkl3 *gene, the following were determined: 94% similarity with *Canis familiaris *(dog), 83% similarity with *Rattus norvegicus *(rat), and 90% similarity with *Mus musculus *(mouse). Slightly less similar was the *cox15 *gene with 87% similarity to *Canis familiaris *(dog), 86% similarity to *Rattus norvegicus *(rat), and 86% similarity to *Mus musculus *(mouse). Based on these results, three additional cell lines, HEK-293, CHO, and MDCK were chosen for investigation [18]. The HEK-293 cells were selected to investigate whether or not the genes identified using HeLa cells had functionality in other human-derived cell lines. With significant homology between the two rodent species in terms of gene transcripts, CHO cells were chosen due to their wide-spread use in commercial applications [19]. However, without a central database detailing the sequence of the CHO genome, the existence of homologs for either *cdkl3 *or *cox15 *could not be verified in that species. The canine cell line selected, MDCK, is commonly used in the production of vaccines [19].

When grown under the same spinner flask conditions previously described, two different types of HEK-293 cells (HEK-293 and HEK-293 ACRP30) expressing either *cdkl3 *or *cox15 *grew faster than the control cells (Table 3). The maximum specific growth rate of the HEK-293 cells expressing *cdkl3 *was 19% greater than the control cells; a value statistically significant for α-values as low 0.005. Similarly, the maximum specific growth rate of the HEK-293 cells expressing *cox15 *was 13% greater than the control cells; a value statistically significant for α-values as low 0.01. Cells expressing *cox15 *grew to a maximum viable cell density 9% higher than the control cells; statistically significant for α-values as low 0.005.

HEK-293 ACRP30 cells expressing *cdkl3 *had a maximum specific growth rate 16% higher than the control cells (Table 3); significant for α-values as low 0.005. These cells expressing *cox15 *also had a maximum specific growth rate higher than the control cells, by approximately 14%; statistically significant for α-values as low 0.001. The HEK-293 ACRP30 cells achieved a maximum viable cell density 8% greater than the control cells; statistically significant for α-values as low 0.01.

CHO cells with inserts of either *cdkl3 *or *cox15 *were also found to grow faster than control cells, as shown in Table 3. CHO cells expressing *cdkl3 *had a maximum specific growth rate 9% higher than the control cells; a value statistically significant for α-values as low 0.005. CHO cells expressing *cox15 *had a maximum specific growth rate 5% higher than the control cells; statistically significant for α-values as low 0.025. In addition, cells expressing either *cdkl3 *or *cox15 *achieved maximum viable cell densities 5% (α-value ≥ 0.005) or 7% (α-value ≥ 0.001) greater than the control cells, respectively. In contrast, MDCK cells with inserts of either *cdkl3 *or *cox15 *did not exhibit differences in terms of growth rates or maximum viable cell densities, as shown in Table 3.

### 6. Examining cell proliferation using flow cytometry and ELISA

**Figure 4:**
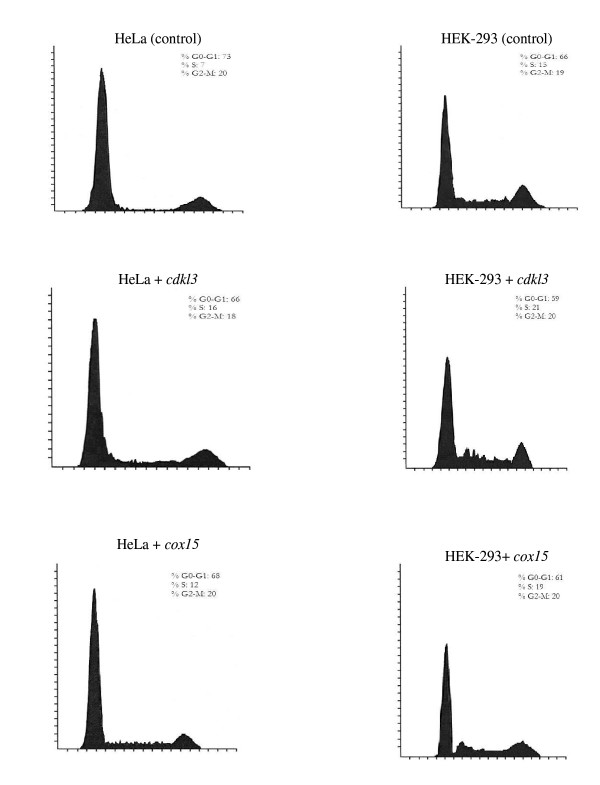
Results of flow cytometric analysis; cell count vs. fluorescence. In each image the first peak (also the largest peak) indicates cells in the G0/G1 phases whereas the second, smaller peak indicates cells in the G2/M phases. In between these two peaks are cells in the S phase. The percentages of cells in each phase are shown along side each figure.

In order to evaluate the effects of enhanced expression of either *cdkl3 *or *cox15 *on cell cycle, analysis of cell cycle progression was performed using flow cytometry. The cell line exhibiting the greatest effects of enhanced expression of either *cdkl3 *or *cox15 *was the attached HeLa cell line. In this assay, cells were initially synchronized and then grown up until late into the exponential phase. Cells with enhanced expression of either *cdkl3 *or *cox15*, shown in Figure 4, illustrated a lower percentage of cells in the GO-G1 phases and a higher percentage in the S phase compared to control cells. The percentage of cells in the G2-M phases was similar between the three cell lines.

To investigate the potential effects of enhanced expression of either *cdkl3 *or *cox15 *on protein production in HEK-293 cells constitutively secreting recombinant adipocyte complement-related protein of 30 kDa (acrp30); HEK-293 ACRP30 were transfected with plasmids containing either *cox15 *or *cdkl3*. The cells were plated in T-flasks at an equivalent seeding density. The total protein production levels in ng/mL were measured in each flask along with the productivities of acrp30 on a per cell basis, shown in Table 4. Cells were assayed during the exponential growth phase and not during the stationary phase, therefore the cell densities listed were not maximum. The total protein levels were increased by 20% in the cells transfected with *cdkl3 *while an 11% increase was seen in cells transfected with *cox15*. Specific production levels were higher in the cells expressing either *cdkl3 *or *cox15 *relative to control cells.

**Table 4:** ELISA results for HEK-293 ACRP30 cells with and without gene inserts. The results shown are from 1 of 3 different runs performed, but are representative of all 3 runs

Cell line	Viable cell density (10^6 ^cells/mL)	Total protein production (ng)	Average specific protein production (ng⋅mLcells MathType@MTEF@5@5@+=feaafiart1ev1aaatCvAUfKttLearuWrP9MDH5MBPbIqV92AaeXatLxBI9gBaebbnrfifHhDYfgasaacH8akY=wiFfYdH8Gipec8Eeeu0xXdbba9frFj0=OqFfea0dXdd9vqai=hGuQ8kuc9pgc9s8qqaq=dirpe0xb9q8qiLsFr0=vr0=vr0dc8meaabaqaciaacaGaaeqabaqabeGadaaakeaadaWcaaqaaiabb6gaUjabbEgaNjabgwSixlabb2gaTjabbYeambqaaiabbogaJjabbwgaLjabbYgaSjabbYgaSjabbohaZbaaaaa@3B07@)
control with plasmid	1.24 ± 0.01	1580 ± 130	1.27 × 10^-3^
+ *cdkl3*	1.40 ± 0.02	1900 ± 160	1.36 × 10^-3^
+ *cox15*	1.31 ± 0.03	1750 ± 190	1.34 × 10^-3^

Note: Each cDNA slide analyzed had at least 3 spots for each of the genes listed above. Analysis of 4 slides translated to a total of at least 12 ratios for each gene. Both the maximum and the minimum expression ratios per gene were determined from these 12 ratios (i.e. sample size).

## Discussion

Using clustering algorithms driven by gene ontology, differentially expressed genes with functionalities relevant to cell growth were identified when comparing slow growing, attached HeLa cells to fast growing, suspension HeLa cells (Table 1). Two of the genes selected for further investigation, *cdkl3 *and *cox15*, were expressed at higher levels in the suspension than in the attached HeLa cells, though each gene was thought to relate to cellular growth in a distinct way. Previous studies suggest *cdkl3 *(cyclin-dependent kinase like 3) is involved in cell cycle regulation/progression, and *cox15 *(cytochrome c oxidase assembly protein) is involved in energy metabolism [20,21].

The gene *cdkl3 *encodes a polypeptide of 455 amino acids titled NKIAMRE [Swiss-Prot: Q8IVW4] that localizes in the cytoplasm and is thought to be a component of a kinase complex that phosphorylates the C-terminus of RNA polymerase II [20,22,23]. Although, the precise functionality of NKIAMRE remains unknown, sequence similarities and the presence of several motifs together imply membership to both the mitogen-activated protein kinase (mapk) family and cyclin-dependent kinase (cdk) family [20,24]. Both of these families are known to be involved in signaling, cell cycle regulation, migration, and survival [25].

As the gene name suggests, *cdkl3 *is most similar in sequence to cyclin-dependent kinase 3 (*cdk3*); a gene that encodes a kinase believed to be necessary for the G1-S transition in mammalian cells [10,26]. The protein encoded by *cdkl3 *also contains the highly conserved TXY (Threonine-X-Tyrosine) motif found in the activation loop domains of either a mapk or cdk [20,24]. In addition, two residues, a serine and a tyrosine, in subdomain I closely resemble an adenosine triphosphate (ATP) binding domain and a negative regulation site typical of a cdk. The protein's name itself, NKIAMRE signifies an amino acid sequence thought to be a cyclin-binding domain also found in a cdk [20,22]. Additionally, the catalytic domain of NKIAMRE contains two highly conserved sequences found in both serine/threonine and tyrosine protein kinases [20,24]. High expression of *nkiamre *was detected in two aggressive types of anaplastic large cell lymphoma (ALCL) tumors when compared to peripheral blood lymphocytes [27]. This finding suggests the gene may also play a role in pathogenesis and/or tumorigenesis.

The gene *cdkl3 *appears to act as either a cell cycle mimic or regulator with several distinct functions including ATP & nucleotide binding, kinase activity, and transferase activity; all of which relate to regulation of the cell cycle and control of cellular proliferation. The current study supports these claims and suggests that *cdkl3*, like its homolog *cdk3*, influences the cell cycle through observations of increased growth rates, reduced lag phases, and greater protein production. The gene *cdk3 *is thought to be rate-limiting for mammalian cell cycle progression [28]. Other studies have shown mammalian cells require *cdk3 *to exit the G0 and G1 phases as well as enter the S phase [29,30]. These observations are also supported by the present work through flow cytometry experiments that indicated cells with an insert of *cdkl3 *were able to transition from the G0/G1 phases to the S phase faster than cells without the insert.

The human gene *cox15 *could relate to cellular growth from the perspective of energy metabolism (i.e. cells growing faster have greater energy demands). It encodes a cytochrome c oxidase (COX) assembly protein referred to as COX15 [Swiss-Prot: Q7KZN9] which localizes in the mitochondrial inner membrane [31,32]. Significant expression of *cox15 *has been observed in tissues with high oxidative phosphorylation demands such as muscle, heart, and brain [21,32].

Acting as the terminal enzyme of the respiratory chain, COX is responsible for the transfer of electrons to molecular oxygen, contributing to the generation of ATP via the proton motive force [31,33]. COX15 is one of several accessory proteins not part of the overall complex, but relevant to the structure, synthesis, and processing of COX [32,34]. Because COX is a large and intricate complex that is continually being used, assembly proteins are also thought to regulate its activity [32,35]. COX is thought to be one of the rate-controlling steps of oxidative phosphorylation [36]. Together, these ideas suggest higher expression of *cox15 *could improve the efficiency of respiration. Support for this can be found in the present study in that cells with an insert of *cox15 *grew faster and produced more recombinant protein (acrp30) than cells without the insert.

Previous studies have shown COX15 is involved in the synthesis of heme a, a compound believed to be critical to energy conservation during oxygen reduction by COX [33,37,38]. In fact, defects in the *cox1*5 gene have resulted in heme a deficiencies whereas defects in COX function have lead to the onset of degenerative diseases such as Leigh syndrome and encephalohepatopathy [21,32,35]. To date, only a few cases of these diseases have been the result of a *cox15 *mutation; and in those select cases the mutations were heterogeneous suggesting the loss of functional COX15 may be fatal [21,34]. It has been proposed that compounds like heme a when bound to proteins, can facilitate the removal of damaging and apoptosis-triggering oxygen radicals [39,40]. These observations and proposals suggest that higher *cox15 *expression might counteract apoptosis, bolstering cell viability. This notion is consistent with findings of the present study where insertion of *cox15 *resulted in higher viable cell densities than control cells.

The present study has shown the value of comparing fast growing, suspension cells with the slow growing, attached cells using microarrays to uncover genes that may contribute to the enhanced cell growth and improved survival of attached HeLa cells. Two genes were found relevant to enhanced cellular growth and increased viabilities; *cdkl3 *and *cox15*. The proposed functions for these genes, obtained from previous studies, are in agreement with the changes that were observed when over-expressed in several mammalian cell lines. The ability to control the cell cycle and apoptosis can be useful in cell engineering for biotechnology applications by providing greater cell mass and improved yields of recombinant protein [1,3]. The comparative physiology studies shown here, provide a useful tool for identifying genes that may serve critical roles in cell growth and other cellular functions. In the present work, a handful of genes were investigated separately, but the potential of this approach (using genomics tools to engineer cell lines) may rely on being able to modulate the expression of many genes simultaneously, rather than individual genes. As such, the present study represents an initial step attempting to directly control cellular features with specific objectives in mind.

## Methods

### 1. Bioreactor setup and sampling

The two HeLa cell lines, attached and suspension were obtained from the American Type Culture Collection (ATCC, Manassas, VA) (Catalog Nos. CCL-2 and CCL-2.2, respectively). Each cell line was grown in a BioFlo 3000 bioreactor (New Brunswick Scientific Co., Edison, NJ) with a working volume of 1.5 L. Both cells lines were grown concurrently. Runs were conducted for up to 7 days after inoculation with constant sampling to characterize growth parameters. Three different runs were carried out for each cell line. The media used was DMEM (Biosource International, Camarillo, CA) supplemented with 10% FBS (Biosource International, Camarillo, CA). The attached HeLa cells were grown on Cytodex 3 (Amersham Biosciences, Piscataway, NJ) microcarriers. Each reactor was seeded with 2.0 × 10^5 ^cells/mL [41,42]. Cells used to seed the bioreactors were synchronized using serum deprivation for 24 hours [6,15].

Each bioreactor was sampled at regular intervals to test media composition (i.e. pH, Glucose, Lactate), cell viability, and cell density. Each bioreactor was sampled for microarray analysis at the same time corresponding to different phases of growth under batch conditions. Samples were placed in 2 mL RNase/DNase-free micro tubes (Marsh Biomediacal Products, Rochester, NY), combined with TRIzol reagent (Invitrogen, Carlsbad, CA) and stored at -80°C.

### 2. RNA isolation and sample preparation

Total RNA was purified from samples using an Invitrogen kit (Micro-to-Midi Total RNA Purification System) and quantified using a spectrophotometer, GeneQuant Pro (Biochrom Ltd, Cambridge, UK). Absorbance values at 260 nm and the other at 280 nm were determined. The quality of RNA was determined by the ratio of these numbers (A_260/280_). Only samples with an A_260/280 _of at least 1.8 were subsequently used for microarray analysis [15]. Each sample, consisting of approximately 10 μg of total RNA, was reverse transcribed, labeled, and prepared for microarray hybridization using a protocol from The Institute for Genomic Research (TIGR) in Rockville, MD: "Aminoallyl labeling of RNA for Microarrays" (SOP # M004, Rev. 2) with an effective date of 3/4/2002.

### 3. cDNA microarray analysis

Microscope slides (Corning, Corning, NY) were printed with a set of 32,448 spots corresponding to approximately 14,000 unique genes by TIGR (Rockville, MD) using an array fabricator (Intelligent Automation, Rockville, MD). Microarray preparation and hybridization were performed using a protocol "Microarray labeled probe hybridization" (SOP # M005, Rev. 3) available from TIGR with an effective date of 9/11/2002. After washing and drying, each slide was ready for scanning and image analysis using a GenePix 4000B (Molecular Devices Corporation, Sunnyvale, CA). Both technical and biological replicates were included in the microarray experiments. To ensure proper labeling efficiency the dyes for half of the arrays were swapped [13,15,43].

Acuity software (Molecular Devices Corporation, Sunnyvale, CA) was used to analysis the data starting with total intensity normalization [13]. Additional steps were taken to filter the data, removing spots with either poor signal quality or genes with highly variable (i.e. inconsistent) expression ratios [43]. The expression ratio for a gene, as referred to in the present study, is defined as the test sample (suspension cell line) intensity divided by the control sample (attached cell line) intensity. An expression ratio of unity indicates equal hybridization between the two samples being assayed whereas expression ratios above or below 1.0 are referred to as being upregulated or downregulated, respectively [14].

The clustering algorithms applied to the data included: self-organizing maps (SOMs), principle component analysis (PCA), and hierarchical clustering [14]. Genes clustered together were probed to identify subsets of genes with relevance to cellular growth (i.e. cell cycle regulation, apoptosis, and/or signal transduction). The most differentially expressed were then identified and evaluated based on their known or proposed functionalities as indicated in the literature [13,15].

Microarray results were verified by performing reverse transcription-polymerase chain reaction (RT-PCR) experiments. The protocols used were provided by Applied Biosystems (4333458, Rev. B & 4335626, Rev. C, both dated 05/2004). Each gene was assayed three times through two separate experiments to establish greater statistical significance.

### 4. Enhanced expression of genes in various cell lines

Sequencing information for both *cdkl3 *and *cox15 *were obtained using two public online databases; Harvester (European Molecular Biology Laboratory, Heidelberg, Germany) and GenBank (National Institutes of Health, Bethesda, MD) [16,17]. For each gene, a plasmid containing the full-length cDNA was purchased from GeneCopoeia (Germantown, MD). Each plasmid also contained the neomycin gene, conferring resistance to the compound geneticin (Invitrogen, Carlsbad, CA). This resistance allowed for the selection of clones expressing the plasmid in the mammalian cell lines examined [5,10]. In subsequent experiments, cells transfected with empty plasmids (i.e. plasmids containing the neomycin gene and other components but neither genes of interest, *cdkl3 *or *cox15*) served as the control.

To check gene and protein sequence homologies the Basic Local Alignment Search Tool (BLAST) provided by the National Center for Biotechnology Information (NCBI) (Bethesda, MD) was used. Based on these results, several cell lines were selected for investigation. Each of the following cell lines was purchased from ATCC (Manassas, VA): HEK-293 (Catalog No. CRL-1573), CHO (Catalog No. CCL-61), and MDCK (Catalog No. CCL-34). Another cell line, HEK-293 ACRP30, was also studied because it constitutively expresses acrp30 for which an enzyme-linked immunosorbent assay (ELISA) is available [44,45]. This cell line was included in subsequent studies to evaluate the impact of cdkl3 and cox15 on protein production. Both HEK-293 cell lines were grown in DMEM (Biosource International, Camarillo, CA) with 10% FBS (Biosource International, Camarillo, CA). The media used with the CHO cells was F-12K (ATCC, Manassas, VA) supplemented with 10% FBS (ATCC, Manassas, VA). The MDCK cells were grown in EMEM (ATCC, Manassas, VA) supplemented with 10% FBS (ATCC, Manassas, VA). All of these cell lines were grown in an incubator (Thermo Scientific, Waltham, MA) set at 37°C and 5% CO_2_. In addition, all of the cell lines were dissociated using a trypsin-EDTA solution (ATCC, Manassas, VA).

Once several different cell lines were selected for analysis, subsequent transfections were performed using manufacturer provided protocols packaged with each transfecting agent. For the attached HeLa cells and the HEK-293 cell lines (HEK-293 and HEK-293 ACRP30), the transfecting agent used was Lipofectamine 2000 (Invitrogen, Carlsbad, CA). In contrast, the transfecting agent used for the suspension HeLa cells and the CHO cells was Lipofectamine LTX (Invitrogen, Carlsbad, CA). And for the MDCK cells, Optifect (Invitrogen, Carlsbad, CA) was the transfecting agent used. The transfecting agents were selected based on consultation with the manufacturer, Invitrogen (Carlsbad, CA) in order to maintain consistent transfection efficiency levels between cell lines and have reproducible results.

Transfections were performed in 24-well plates (Corning, Corning, NY). Within 24 hours following the transfection the media was replaced with complete media. Once the cells had recovered, usually after 48 hours, geneticin was added to a final concentration of 750 μg/mL based on a prior kill curves (data not shown). Over several days, as media was periodically replaced, colonies began to form. Individual colonies were isolated and moved into 96-well plates (Corning, Corning, NY). Upon reaching confluency, each well was expanded sequentially into 24, 12, and 6-well plates (Corning, Corning, NY) before being counted and screened for expression based on western blot analysis. Physiological changes, post-transfection, were also observed using a DM IRB microscope and attached camera (Leica Camera, Allendale, NJ).

Western blotting was conducted to verify translation of gene inserts via transfected plasmids. Protocols for this method were made available by the manufacturers of the primary antibodies for COX15 (Abnova, Taipei City, Taiwan) and CDKL3 (Abcam, Cambridge, MA). Briefly, cell lysate samples were prepared from a population of cells that were counted and assayed for viability. These samples were diluted with reducing sample buffer and separated using 4–20% Tris-Glycine gels (Invitrogen, Carlsbad, CA). Proteins were transferred to nitrocellulose membranes (Invitrogen, Carlsbad, CA) and blocked with blotting grade blocker, non-fat dry milk (Bio-Rad, Hercules, CA) for 1 hour. Membranes were then incubated with the primary antibodies at a dilution of 1:500 for 1 hour followed by incubation with secondary antibodies (HRP-conjugated secondary antibodies) for another hour. Incubation with a color development agent (KPL, Gaithersburg, MD) allowed visualization of the blots which were then scanned.

Following clone screening, cells expressing the desired plasmid were expanded successively into 6-well plates and 25 cm^2 ^T-flasks. For growth comparisons, 75 cm^2 ^T-flasks (Corning, Corning, NY) and 250 mL spinner flasks (Bellco Glass, Vineland, NJ) were seeded and monitored for pH (Radiometer Analytical SAS, Lyon, France), cell viability & density (Cedex HiRes, Innovatis, Malvern, PA), and metabolite concentrations (YSI, Yellow Springs, OH) [41,42]. Cell counting was performed two different ways; using a cell counter (Cedex HiRes) and using a hemocytometer. In both cases, duplicates of each sample were assayed. The Cedex HiRes was used as instructed by the manufacturer. Samples counted manually were diluted 1:1 with tryphan blue and counted on a hemocytometer. Variation between duplicates and between counting methods were calculated to be = 2.5% In conjunction with the spinner flasks, Cytodex 3 microcarriers (GE Healthcare, Piscataway, NJ) were used for the cell lines studied [13]. Cells used for seeding were synchronized using the mitotic shake-off method [6,9].

### 5. Flow cytometry and ELISA

Cells grown in 162 cm^2 ^T-flasks were sampled at different points along their respective growth curves for analysis using a flow cytometer. Samples were taken during the early, middle, and late exponential growth phase, following synchronization by the mitotic shake-off method [6,9]. Flow cytometry experiments were performed using a CyAn LX flow cytometer (DakoCytomation, Fort Collins, CO). Cells were fixed with ethanol and stained with propidium iodide (Becton Dickinson, Franklin Lakes, NJ) at a concentration between 0.5 – 1.0 × 10^6 ^cells/mL [46]. Summit software (DakoCytomation, Fort Collins, CO) was used to capture data generated by the sampled cells such as the level of light scattering and fluorescence. In contrast, Modfit software (Molsoft, La Jolla, CA) was used to identify varying stages of the cell cycle and calculate the percentages of cells in each stage.

To quantify the amount of acrp30 secreted by HEK-293 ACRP30 cells an ELISA was performed using a kit produced by R&D Systems (Catalog No. MRP300, Minneapolis, MN). A microplate spectrophotometer (SpectraMax 190, Molecular Devices, Sunnyvale, CA) was used to quantify the amount of protein present in the samples. Samples were run at varying dilutions to establish the minimum dilution level necessary to obtain values that fall within the standard curve. This value was determined to 1:4,000 (data not shown).

## Authors' contributions

PJ was involved in the conception of the work, performed many of the experiments, and drafted portions of the manuscript. MB conceived part of the work and helped draft the manuscript. KK was involved in the microarray experiments and drafting the manuscript. JS designed several experiments, coordinated much of the work, and drafted portions of the text. All authors read and approved the final manuscript.
